# Serum CXCL1 Is a Prognostic Factor for Patients With Hepatitis B Virus–Related Acute-On-Chronic Liver Failure

**DOI:** 10.3389/fmed.2021.657076

**Published:** 2021-07-28

**Authors:** Lanlan Xiao, Shima Tang, Lingjian Zhang, Shanshan Ma, Yalei Zhao, Fen Zhang, Zhongyang Xie, Lanjuan Li

**Affiliations:** State Key Laboratory for Diagnosis and Treatment of Infectious Diseases, National Clinical Research Center for Infectious Diseases, Collaborative Innovation Center for Diagnosis and Treatment of Infectious Diseases, College of Medicine, The First Affiliated Hospital, Zhejiang University, Hangzhou, China

**Keywords:** CXCL1, HBV-ACLF, neutrophils, inflammation, chemokine

## Abstract

**Purpose:** Neutrophils and cytokines play a major role in the pathogenesis of acute-on-chronic liver failure (ACLF). We aimed to determine whether chemokine (CXC) ligand 1 (CXCL1), a key marker of neutrophil recruitment and activation, could predict the severity and prognosis of hepatitis B virus–related ACLF (HBV-ACLF).

**Methods:** Hospitalized patients with HBV-ACLF were enrolled in a prospective study and stratified as survivors (alive at 28 days) and nonsurvivors (deceased at 28 days). Serum CXCL1 levels were measured in healthy controls, patients with chronic HBV, patients with HBV-related compensated cirrhosis, and patients with HBV-ACLF. Univariate and multivariable logistic analyses, Pearson correlation analysis, area under the receiver operating characteristic curve (AUROC), and *Z* tests were used to evaluate the performance of CXCL1 as a marker in HBV-ACLF.

**Results:** Patients with HBV-ACLF had significantly higher serum levels of CXCL1 and neutrophil count than healthy controls and patients with chronic HBV or HBV-related compensated cirrhosis (*P* < 0.01, respectively). Among patients with HBV-ACLF, survivors had lower serum CXCL1 levels and neutrophil count than those of nonsurvivors (*P* < 0.001, *P* < 0.05, respectively). Serum CXCL1 level was positively correlated with neutrophil count (*r* = 0.256, *P* = 0.001), ACLF grade (*r* = 0.295, *P* < 0.001) and organ failure, including coagulation (*r* = 0.21, *P* = 0.005) and brain failure (*r* = 0.198, *P* = 0.008). Multivariable logistic analyses showed serum CXCL1 [OR (95% CI) = 1.017 (1.009–1.025), *P* < 0.001] was an independent risk factor for 28-day mortality in HBV-ACLF. Meanwhile, the AUROC analysis demonstrated that serum CXCL1 [0.741 (0.669–0.804)] might be a reliable prognostic biomarker for patients with HBV-ACLF.

**Conclusions:** Overall, serum CXCL1 can serve as a biomarker indicating the severity of disease and prognosis for patients with HBV-ACLF. CXCL1 might also be a therapeutic target in this disease.

## Introduction

Acute-on-chronic liver failure (ACLF) is a complex syndrome characterized by acute deterioration of liver function in patients with pre-existing chronic liver disease (1). An estimated 240 million people worldwide are chronically infected with the hepatitis B virus (HBV), 75% of whom in Asia (2, 3), causing HBV infection become the major etiology for ACLF in the Asia-Pacific region, with HBV-related ACLF (HBV-ACLF) accounting for over 70% of ACLF cases (4, 5). ACLF usually progresses rapidly and has a mortality rate of more than 50%, even after aggressive treatments (6–8). Therefore, early diagnosis and prediction of outcome are critical for improving survival.

Neutrophils are considered to be one of the main effectors during acute inflammation (9). Hepatic infiltration by neutrophils and their subsequent activation are a rapid response to sterile and nonsterile tissue injury (10). Studies demostrated that neutrophils and proinflammatory cytokines have been implicated in the pathogenesis of acute and chronic liver injury (11). Chemokine (CXC) ligand 1 (CXCL1), which belongs to the CXC chemokine family, is the key chemokine for neutrophil infiltration and activation, and mediates neutrophil recruitment through binding to CXC receptor 2 (CXCR2) (12, 13). Mice overexpressing CXCL1 in their lungs have augmented neutrophil influx, whereas blockade of this chemokine with neutralizing antibodies markedly blunts neutrophil recruitment (13). Evidences showed that CXCL1 orchestrates neutrophil-dependent immunity–induced lung inflammation, sepsis, colitis, and cancer (13). In addition to its role in inflammation, CXCL1 also has an important role in angiogenesis, tumorigenesis, and wound healing (13).

Recently, an increasing number of studies have demonstrated that CXCL1 is involved in pathogenesis in liver disease, including in alcoholic liver injury, nonalcoholic steatohepatitis (NASH), and hepatocellular carcinoma (HCC) (14–16). However, little is known about the clinical relevance of CXCL1 in HBV-ACLF. In a prospective study, we investigated serum levels of CXCL1, finding that CXCL1 may potentially be used to predict severity and prognosis of HBV-ACLF.

## Materials and Methods

### Patients

Ethical approval was obtained from the Ethics Committee of the First Affiliated Hospital, Zhejiang University School of Medicine, and informed consent was obtained from all participants included in this prospective study. Between April 2018 and August 2020, 25 eligible healthy controls, 24 outpatients with chronic hepatitis B (CHB), 25 outpatients with HBV-related compensated cirrhosis (HBV-CC), and 175 inpatients with HBV-ACLF from the First Affiliated Hospital of Zhejiang University were enrolled in the study.

**Figure 1:**
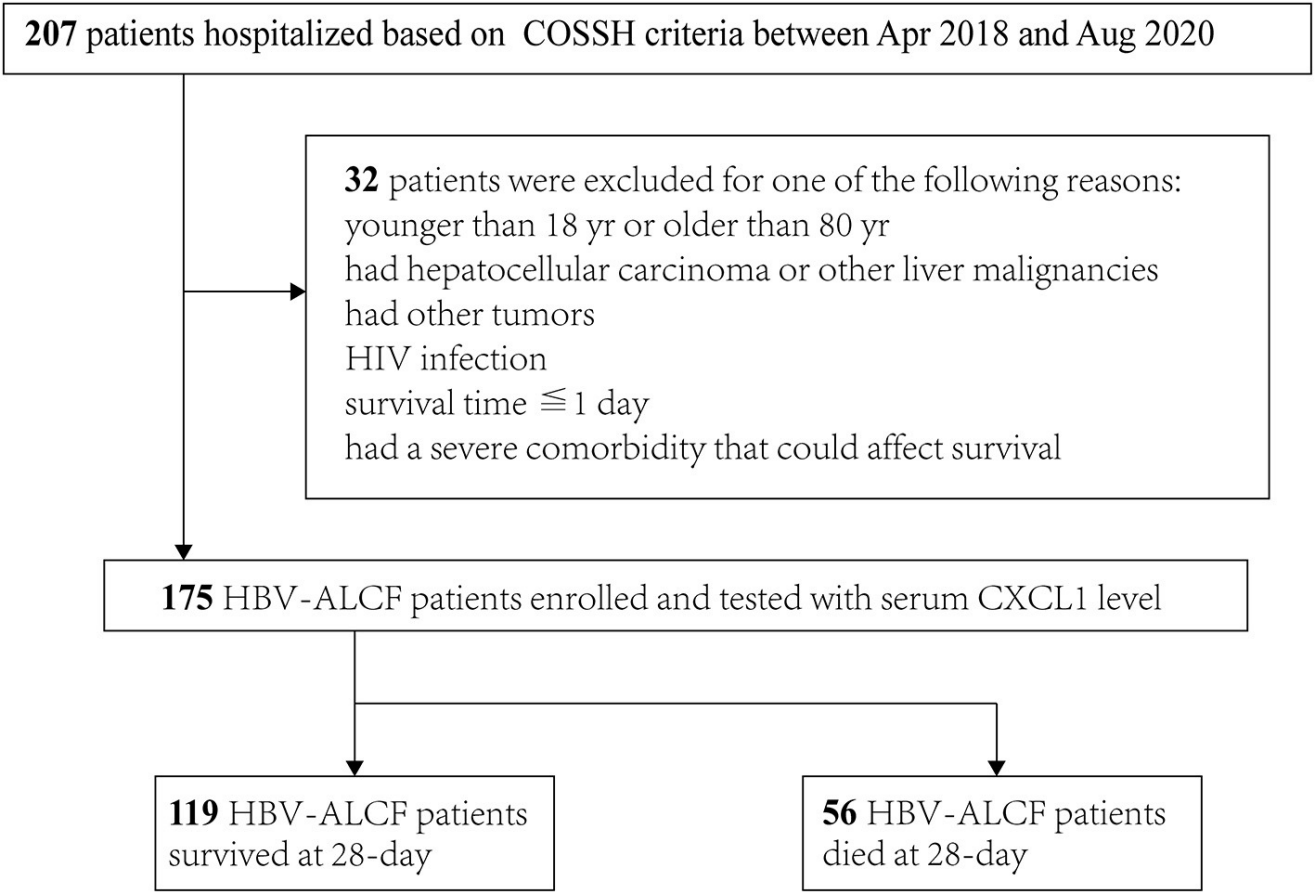
Flowchart of the patient selection process. COSSH-ACLF score, Chinese Group on the Study of Severe Hepatitis B-ACLF; HIV, human immunodeficiency virus; HBV-ACLF, hepatitis B virus-related acute-on-chronic liver failure; CXCL1, C-X-C motif chemokine ligand 1.

The hospitalized patients with HBV-ACLF were screened according to criteria provided by the Chinese Group on the Study of Severe Hepatitis B–ACLF (COSSH-ACLF) (17) at admission. Exclusion criteria included (1) age < 18 or > 80 years; (2) a history of HCC or other liver malignancies; (3) a history of other tumors; (4) human immunodeficiency virus (HIV) infection; (5) survival time of ≤ 1 day following admission; and (6) presence of a severe comorbidity that could affect survival. Patients with HBV-ACLF were stratified on the basis of 28-day survival into survivors and nonsurvivors (Figure 1). HBV-CC patients without previous major complications, including ascites, hepatic encephalopathy (HE), gastrointestinal hemorrhage, spontaneous bacterial peritonitis (SBP), hepatorenal syndrome, portopulmonary hypertension (18). CHB and liver cirrhosis were diagnosed according to criteria outlined in previous reports (19, 20). HE was defined and graded using the West Haven criteria (21).

### Management of HBV-ACLF

All patients with HBV-ACLF received standard medical treatment to eliminate or control precipitating factors and associated complications during hospitalization (22, 23), including bed rest, nutritional support, maintenance of water/electrolyte balance, and antiviral therapy with nucleoside analogs; sodium restriction and diuretics for ascites; l-ornithine aspartate, lactulose, and antibiotics for HE; prophylactic antibiotics for bacterial infection; oxygen therapy for respiratory dysfunction; and renal replacement therapy for hepatorenal syndrome and uremic symptoms. Artificial liver support system and liver transplant were considered when the above measures failed. Extracorporal liver support system and liver transplant were considered for some patients.

### Data and Sample Collection

We collected the following clinical and demographic information for participants: demographic data, incidence of complications, laboratory measurements [e.g., serum albumin, sodium, alanine aminotransferase (ALT), aspartate aminotransferase (AST), total bilirubin (TBIL), neutrophil count, international normalized ratio (INR), and creatinine levels, HBV DNA abundance, etc.], mean arterial pressure, pulse oximetry, events of organ failure, and prognosis. Because the primary endpoint of the study was 28-day mortality, all patients with HBV-ACLF were followed for at least 28 days (by telephone and outpatient record enquiry). Patients receiving a liver transplant within 28 days were included in the nonsurvivor group. Blood samples were obtained at admission and subsequently centrifuged at 4°C for 10 min at 3,500 rpm.; the serum samples obtained were stored at −80°C.

### Severity Assessment and Prognostic Models

ACLF grade was determined based on the COSSH-ACLF criteria (17) as follows. ACLF grade 1 included four subgroups: (1) kidney failure alone; (2) single liver failure with INR ≥ 1.5 and/or kidney dysfunction and/or HE grade I or II; (3) single type of organ failure, including coagulation or circulatory or respiratory system failure, and/or kidney dysfunction and/or HE grade I or II; and (4) cerebral failure alone together with kidney dysfunction. ACLF grade 2 was defined as failure of two organs. ACLF grade 3 was defined as failure of three or more organs.

Organ failure was diagnosed using the CLIF Consortium Organ Failure Score (CLIF-C OFs) as described previously. Briefy, liver failure (TBil ≥ 12 mg/dL), kidney failure (serum creatinine ≥ 2 mg/dL), brain failure (grade III–IV HE), coagulation failure (INR ≥ 2.5), circulation failure (use of vasopressors), and lung failure (PaO2/FiO2 ≤ 200 or SpO2/FiO2 ≤ 214) (24). Prognostic scoring systems were used to predict 28-day mortality for patients with HBV-ACLF, including Model for End-Stage Liver Disease (MELD), MELD-sodium (MELD-Na), integrated MELD (iMELD), CLIF-C OFs, CLIF Consortium ACLF score (CLIF-C ACLFs), and COSSH-ACLF score (COSSH-ACLFs). The scores were calculated as detailed in the Supplementary Material.

### Measurement of Serum CXCL1

CXCL1 levels were assayed by enzyme-linked immunosorbent assay (ELISA) with a human CXCL1 ELISA kit (Abcam). Assays were performed in accordance with the manufacturer's guidelines. Briefly, Standard was Prepared from 0 to 150 pg/ml, 50 μL sample serum and Standard was added to Anti-tag coated microplate. Then 50 μL of the Antibody Cocktail to each well, gently mixed, and incubated for 1 h at room temperature. Subsequently, wash each well with Wash Buffer, add 100 μL of TMB Development Solution to each well and incubate for 10 min in the dark. Finally, add 100 μL of Stop Solution to each well and record the OD at 450 nm using a spectrophotometer (Epoch 2 Microplate Spectrophotometer). The sensitivity of the kit was 0.064 pg/ml, and inter- and intra-assay assessments of kit reliability were also performed.

### Statistical Analysis

Categorical data are presented as numbers (percentages) and were compared using chi-squared or Fisher's exact tests. Distributions of continuous data are shown as the mean ± standard deviation and were compared by Student's *t*-test (normal continuous variables) or Wilcoxon rank-sum test (non-normal continuous variables). Pearson correlation analysis was performed to identify correlation between CXCL1 levels and other clinical parameters. Univariate and multivariate regression analysis. were used to identify independent predictors of short-term mortality. Area Under the Receiver Operating Characteristic curve (AUROC) values were calculated, and *Z* tests (Delong's method) (25) were used to compare the predictive value of different prognostic scoring systems. Statistical analyses were performed using SPSS (version 18.0, SPSS). *P* < 0.05 was considered to indicate statistical significance.

## Results

### Characteristics of the Enrolled Participants

Between April 2018 and August 2020, 207 inpatients with HBV-ACLF were screened. Of these, 175 eligible patients with HBV-ACLF were enrolled, of whom 119 were included in the survivor group and 56 were included in the nonsurvivor group (Figure 1). The majority of patients were male in both groups (82.4 vs. 85.7%, respectively). Nonsurvivors were older (52.1 ± 11.7 vs. 46.0 ± 12.2 years, *P* = 0.002). The admission condition of nonsurvivors was worse than that of survivors, with nonsurvivors having a higher frequency of HE (*P* < 0.01), organ failure (coagulation and brain failure, *P* < 0.001 and *P* = 0.003, respectively), and ACLF grade 2 or 3 (*P* < 0.001 for both). Levels of AST (297.0 ± 315.3 U/L vs. 182.7 ± 169.1 U/L, *P* = 0.012), TBIL (384.1 ± 148.6 μmol/L vs. 337.4 ± 103.8 μmol/L, *P* = 0.034), prothrombin time (30.4 ± 10.9 s vs. 21.6 ± 5.6 s, *P* < 0.001), and INR (2.7 ± 1.1 vs. 1.9 ± 0.5, *P* < 0.001) were also significantly higher in nonsurvivors than in survivors. The platelet count were lower in nonsurvivors than survivors (92.1 ± 51.6 × 10^9^/L vs. 108.5 ± 49.6 × 10^9^/L, *P* = 0.043). Nonsurvivors had higher MELD, MELD-Na, iMELD, CLIF-C OFs, CLIF-C ACLFs, and COSSH-ACLFs scores than those of survivors (*P* < 0.001 for all comparisons). The characteristics of all participants are listed in Table 1.

**Table 1:** Comparison of demographic and clinical characteristics of subjects.

**Characteristics**	**Healthy controls** **(*N* = 25)**	**Chronic hepatitis B (*N* = 24)**	**HBV-CC** **(*N* = 25)**	**HBV-ACLCF (** ***N*** **=** **175)**		***P***
				**Survivors** **(*N* = 119)**	**Nonsurvivors** **(*N* = 56)**	
Age (years)	39.4 (13.7)	42.5 (10.9)	52.0 (10.9)	46.0 (12.2)	52.1 (11.7)	0.002
Male sex (no.)	17 (68.0)	17 (70.8)	20 (80.0)	98 (82.4)	48 (85.7)	0.557
Liver cirrhosis (no.)	/	/	/	63 (52.9)	31 (55.4)	0.765
Liver transplantation (no.)	/	/	/	10 (8.4)	20 (35.7)	<0.001
Liver support system (no.)	/	/	/	91 (76.5)	41 (0.641)	0.641
**HBV-DNA level (IU/mL)**						
≤200	/	12 (50)	20 (80)	5 (4.2)	3 (5.4)	0.733
200–2 × 10^4^	/	5 (20.8)	4 (16)	33 (27.7)	12 (21.4)	0.374
2 × 10^4^−2 × 10^6^	/	3 (12.5)	1 (4)	49 (41.2)	20 (35.7)	0.49
≥2 × 10^6^	/	4 (16.7)	0 (0)	32 (26.9)	21 (37.5)	0.154
**Complications (no.)**						
Hypersplenism	/	/	/	25 (44.6)	51 (42.9)	0.824
Spontaneous bacterial peritonitis	/	/	/	10 (8.4)	4 (7.1)	0.774
Gastrointestinal hemorrhage	/	/	/	4 (3.4)	5 (8.9)	0.12
Ascites	/	/	/	63 (52.9)	27 (48.2)	0.559
Infection	/	/	/	11 (9.2)	6 (10.7)	0.759
HE I-II	/	/	/	8 (6.7)	16 (28.6)	<0.001
HE III-IV	/	/	/	3 (2.5)	8 (14.3)	0.003
Laboratory data	/	/	/			
ALT (U/L)	18.3 (7.7)	28.3 (15.0)	29.2 (16.0)	286.8 (321.3)	396.6 (393.5)	0.068
AST (U/L)	18.3 (5.6)	29.0 (18.6)	32.3 (14.0)	182.7 (169.1)	297.0 (315.3)	0.012
ALP (U/L)	66.9 (23.7)	64.3 (21.3)	88.0 (20.8)	144.3 (45.5)	128.5 (43.6)	0.028
Albumin (g/dL)	46.7 (2.8)	44.5 (3.6)	43.9 (4.5)	31.3 (4.3)	31.9 (5.2)	0.436
TBIL(μmol/L)	15.2(16.3)	11.7 (7.7)	26.0 (32.3)	337.4 (103.8)	384.1 (148.6)	0.034
GGT (U/L)	/	/	/	101.4 (77.8)	93.4 (70.0)	0.507
Creatinine (μmol/L)	74.1 (16.5)	75.4 (15.5)	80.5 (20.8)	71.5 (53.5)	81.3 (50.3)	0.249
Sodium (mmol/L)	/	/	/	137.4 (3.7)	137.5 (5.7)	0.995
White blood cell count (10^9^/L)	5.7 (1.4)	5.9 (1.9)	5.6 (2.3)	7.2 (3.1)	7.8 (3.3)	0.235
Neutrophil count (10^9^/L)	3.2 (0.9)	3.3 (1.2)	3.5 (1.6)	5.0 (2.7)	6.0 (2.8)	0.022
Neutrophil percentage (%)	55.8 (8.1)	55.2 (9.8)	62.8 (9.2)	68.4 (10.4)	74.4 (10.7)	0.001
Hemoglobin (g/L)	180.7 (88.0)	147.4 (10.8)	133.5 (15.2)	124.6 (21.2)	120.2 (22.1)	0.208
Platelet count (10^9^/L)	271.8 (169.0)	200.2 (59.1)	154.9 (77.4)	108.5 (49.6)	92.1 (51.6)	0.043
Prothrombin time (s)	/	/	/	21.6 (5.6)	30.4 (10.9)	<0.001
INR	/	/	/	1.9 (0.5)	2.7 (1.1)	<0.001
Procalcitonin (ng/ml)	/	/	/	1.6 (4.8)	2.9 (6.1)	0.185
CXCL1 level (pg/ml)	36.3 (13.4)	32.1 (7.8)	28.0 (10.3)	80.2 (35.5)	143.8 (84.2)	<0.001
Organ failure (no.)						
Liver	/	/	/	106 (89.1)	52 (92.9)	0.431
Coagulation	/	/	/	9 (7.6)	29 (51.8)	<0.001
Kidney	/	/	/	1 (0.8)	0 (0)	0.491
Brain	/	/	/	3 (2.5)	8 (14.3)	0.003
Lung	/	/	/	0 (0)	0 (0)	1
Circulation	/	/	/	0 (0)	0 (0)	1
**Severity score**						
MELD	/	/	/	21.4 (4.7)	26.3 (7.6)	<0.001
MELD-Na	/	/	/	22.1 (5.6)	27.7 (8.2)	<0.001
iMELD	/	/	/	39.2 (6.4)	45.8 (9.6)	<0.001
CLIF-C Ofs	/	/	/	8.6 (1.0)	10.1 (1.4)	<0.001
CLIF-C ACLFs	/	/	/	38.2 (6.0)	47.0 (7.8)	<0.001
COSSH-ACLFs	/	/	/	5.8 (0.7)	7.1 (1.3)	<0.001
**HBV-ACLF (COSSH criteria)**						
ACLF grade 1	/	/	/	108 (90.8)	26 (46.4)	<0.001
ACLF grade 2	/	/	/	10 (8.4)	22 (39.3)	<0.001
ACLF grade 3	/	/	/	1 (0.8)	8 (14.3)	<0.001

*Data are mean ± standard deviation, or number (percentage). P-values for comparisons between **survivors** and nons**urvivors**. HBV-ACLF, hepatitis B virus-related acute-on-chronic liver failure; HE, hepatic encephalopathy; ALT, alanine aminotransferase; AST, aspartate aminotransferase; ALP, alkaline phosphatase; TBIL, total bilirubin; GGT, γ-glutamyl transpeptidase; INR, international normalized ratio; MELD, model for end-stage liver disease; MELD-Na, MELD-sodium; iMELD, integrated MELD; CLIF-C ACLFs, CLIF-consortium-ACLF score; CLIF-C OFs, CLIF-consortium organ failure score; COSSH-ACLF score, chinese group on the study of severe hepatitis B-ACLF*.

### Group Comparison of Serum CXCL1 Levels and Neutrophil Counts

**Figure 2:**
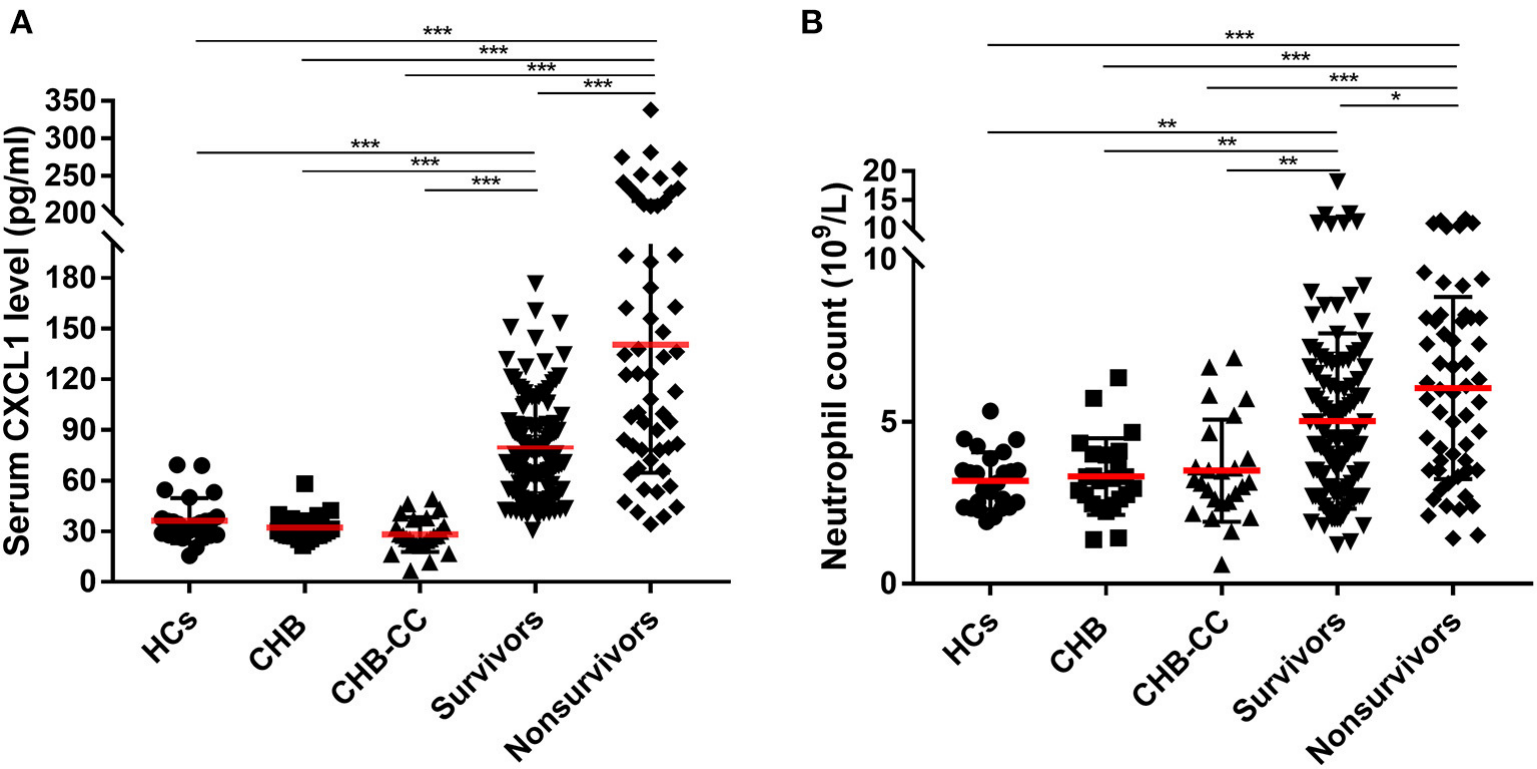
Baseline **(A)** serum CXCL1 level and **(B)** neutrophil count in enrolled subjects. CXCL1, C-X-C motif chemokine ligand 1; HCs, healthy controls; CHB, Chronic Hepatitis B; HBV-CC, HBV-related-compensated-cirrhosis (HBV-CC); survivors, patients with hepatitis B virus-related acute-on-chronic liver failure (HBV-ACLF) survived at 28 days after at admission; nonsurvivors, were patients with HBV-ACLF died at 28 days after at admission. ^*^P < 0.5; ^**^P < 0.01; ^***^P < 0.001.

Baseline serum CXCL1 levels were compared among the groups, including in patients with HBV-ACLF in the nonsurvivor and survivor groups, healthy controls, and in patients with CHB and HBV-CC (Figure 2A). Regardless of the 28-day prognosis, both nonsurvivors (143.8 ± 84.2 pg/ml) and survivors (80.2 ± 35.5 pg/ml) with HBV-ACLF had significantly higher serum levels of CXCL1 than those of healthy controls (36.3 ± 13.4 pg/ml), patients with CHB (32.1 ± 7.8 pg/ml), and patients with HBV-CC (28.0 ± 10.3 pg/ml; *P* < 0.001 for all comparisons; Table 1 and Figure 2A), whereas no statistically significant difference was found when comparing CXCL1 levels in healthy controls and the CHB and HBV-CC groups (*P* > 0.05). Among patients with HBV-ACLF, nonsurvivors had significantly higher serum CXCL1 levels than those of survivors (*P* < 0.001), suggesting that serum CXCL1 levels might be closely associated with 28-day mortality.

Similar results were found in neutrophil count. Patients with HBV-ACLF had significantly higher neutrophil count than those of healthy controls (3.2 ± 0.9 × 10^9^/L), patients with CHB (3.3 ± 1.3 × 10^9^/L), and patients with HBV-CC (3.5 ± 1.6 × 10^9^/L; *P* < 0.01 for all comparisons; Table 1 and Figure 2B). Among patients with HBV-ACLF, nonsurvivors had significantly higher neutrophil count than those of survivors (6.0 ± 2.8 × 10^9^/L vs. 5.0 ± 2.7 × 10^9^/L, *P* < 0.05).

### Serum CXCL1 Is an Independent Risk Factor for Outcome in Patients With HBV-ACLF

To determine the profile of serum CXCL1 levels in patients with HBV-ACLF, we performed analysis assessing the correlation between baseline serum CXCL1 levels and laboratory parameters, organ failure, and prognostic scores (Table 2). We observed that serum CXCL1 levels were inversely correlated with alkaline phosphatase levels (*r* = −0.202, *P* = 0.007), while they were positively correlated with neutrophil count (*r* = 0.256, *P* = 0.001) and prothrombin time (*r* = 0.215, *P* = 0.004). Higher serum CXCL1 levels tended to correlate with organ failure (coagulation: *r* = 0.21, *P* = 0.005; brain failure: *r* = 0.198, *P* = 0.008) and high ACLF grade (*r* = 0.295, *P* < 0.001). Serum CXCL1 levels were also positively correlated with various prognostic scores (MELD: *r* = 0.260, *P* = 0.001; MELD-Na: *r* = 0.294, *P* < 0.001; iMELD: *r* = 0.305, *P* < 0.001; CLIF-C OFs: *r* = 0.307, *P* < 0.001; CLIF-C ACLFs: *r* = 0.324, *P* < 0.001; COSSH-ACLFs: *r* = 0.236, *P* < 0.001).

**Table 2:** Associations of clinical parameters and prognostic scoring systems with serum CXCL1 level in patients with HBV-ACLF.

**Variable**	**Regression coefficient**	***P***
**Laboratory data**		
ALT (U/L)	0.065	0.389
AST (U/L)	0.06	0.425
ALP (U/L)	−0.202	0.007
Albumin (g/dL)	0.017	0.828
White blood cell count (10^9^/L)	0.117	0.122
Neutrophil count (10^9^/L)	0.256	0.001
Prothrombin time (s)	0.215	0.004
Procalcitonin (ng/ml)	−0.013	0.877
HBV-DNA (IU/ml)	0.062	0.416
**Organ failure**		
Liver	0.083	0.273
Coagulation	0.21	0.005
Kidney	0.062	0.417
Cerebral	0.198	0.008
Lung	−0.077	0.431
Circulation	−0.062	0.527
**Severity score**		
MELD	0.26	0.001
MELD-Na	0.294	<0.001
iMELD	0.305	<0.001
CLIF-C OFs	0.307	<0.001
CLIF-C ACLFs	0.324	<0.001
COSSH-ACLFs	0.236	<0.001
ACLF grade (COSSH criteria)	0.295	<0.001

*Data are P-value (r-value). CXCL1, C-X-C motif chemokine ligand 1; HBV-ACLF, hepatitis B virus-related acute-on-chronic liver failure; ALT, alanine aminotransferase; AST, aspartate aminotransferase; ALP, alkaline phosphatase; MELD, Model for End-Stage Liver Disease; MELD-Na, MELD-sodium; iMELD, integrated MELD; CLIF-C ACLFs, CLIF-Consortium-ACLF score; CLIF-C OFs, CLIF-Consortium Organ Failure Score; COSSH-ACLF score, Chinese Group on the Study of Severe Hepatitis B-ACLF*.

Univariate and multivariate regression analyses including serum CXCL1 levels as well as other clinical and laboratory indicators were used to identify independent risk factors for HBV-ACLF 28-day mortality. Multivariate regression analyses showed serum CXCL1 levels had excellent performance [odds ratio (95% confidence interval) = 1.017 (1.009–1.025), *P* < 0.001], implying that serum CXCL1 along with age [odds ratio (95% confidence interval) = 1.041 (1.004–1.081), *P* = 0.31], INR [odds ratio (95% confidence interval) = 5.212 (2.378–11.424), *P* < 0.001] and HE [odds ratio (95% confidence interval) = 2.760 (1.228–6.204), *P* = 0.014; Table 3] might be independent risk factors for HBV-ACLF.

**Table 3:** Univariate and multivariate logistic regression analysis investigating independent risk factors on 28-day survival for patients with HBV-ACLF.

**Variable**	**Reference**	**OR (CI 95 %)**	***P***
**Univariate logistic regression analysis**			
Age (years)	Per year	1.045 (1.017–1.074)	0.002
ALT (U/L)	Per unit increase	1.001 (1.000–1.002)	0.034
AST (U/L)	Per unit increase	1.002 (1.001–1.004)	0.003
Creatinine (μmol/L)	Per unit increase	1.004 (0.997–1.010)	0.232
TBil (μmol/L)	Per unit increase	1.004 (1.001–1.007)	0.005
Neutrophil count (10^9^/L)	Per unit increase	1.139 (1.015–1.278)	0.027
INR	Per unit increase	6.598 (3.337–13.047)	<0.001
HE grade	Per grade increase	4.123 (2.175–7.815)	<0.001
CXCL1 level (pg/ml)	Per unit increase	1.020 (1.013–1.028)	<0.001
**Multivariate logistic regression analysis**			
Age (years)	Per year	1.041 (1.004–1.081)	0.031
INR	Per unit increase	5.212 (2.378–11.424)	<0.001
CXCL1 (pg/ml)	Per unit increase	1.017 (1.009–1.025)	<0.001
HE grade	Per point increase	2.760 (1.228–6.204)	0.014

*HBV-ACLF, hepatitis B virus-related acute-on-chronic liver failure; ALT, alanine aminotransferase; AST, aspartate aminotransferase; TBil, total bilirubin; TNR, international normalized ratio; HE, hepatic encephalopathy; CXCL1, C-X-C motif chemokine ligand 1*.

**Figure 3:**
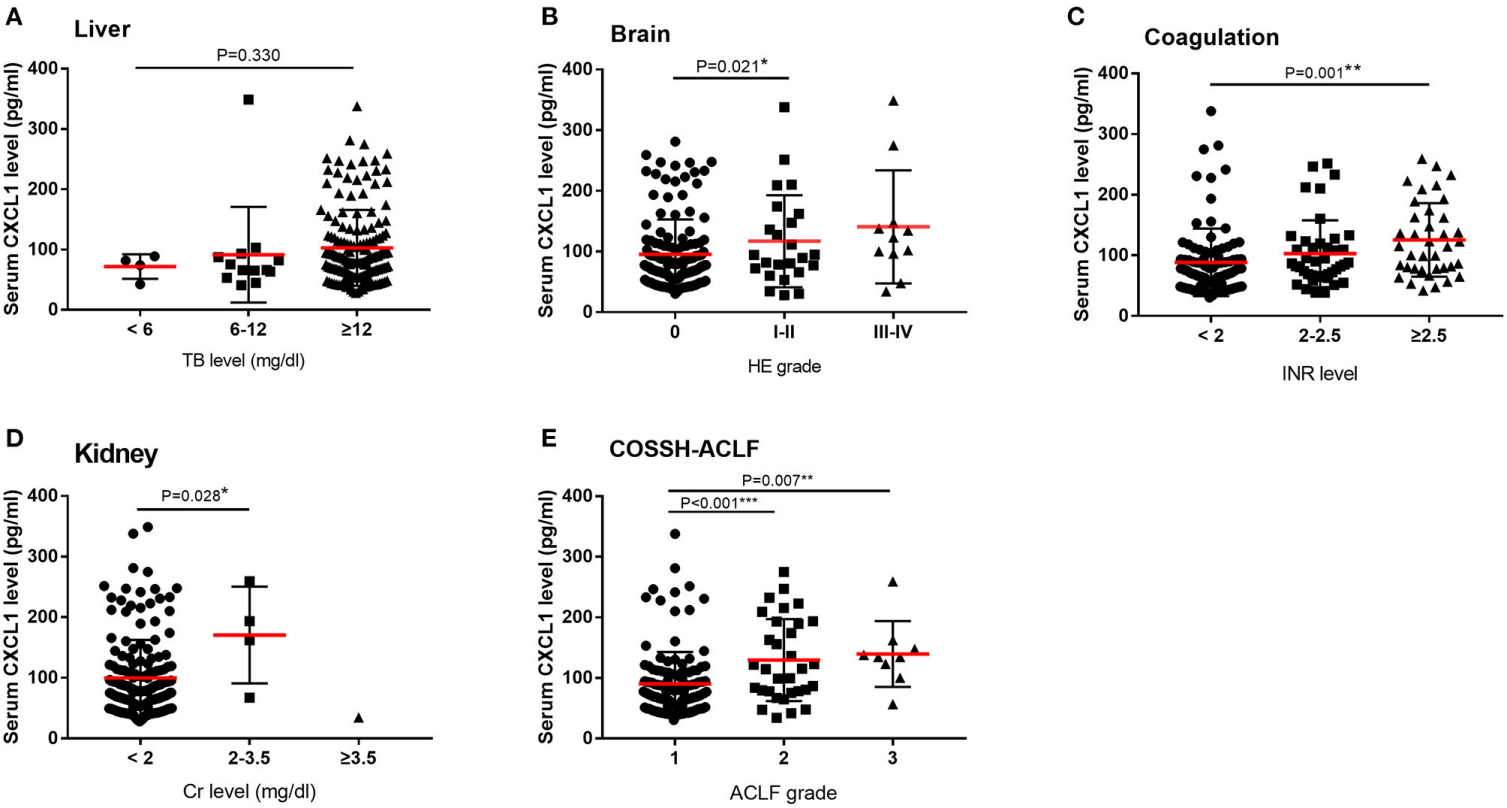
Serum CXCL1 levels and organ injury among patients with HBV-ACLF. Comparisons of serum CXCL1 levels among patients with various degrees of organ injury according to CLIF-OF score and COSSH score. **(A)** liver **(B)** brain **(C)** coagulation **(D)** kidney **(E)** ACLF grade. ^*^P < 0.5; ^**^P < 0.01; ^***^P < 0.001.

We also compared serum CXCL1 levels among patients with various degrees of organ injury, assessed according to CLIF-C OFs. Serum CXCL1 level was positively correlated with brain, coagulation, kidney injury (Figure 3).

### Serum CXCL1 Is a Potential Prognostic Marker for Patients With HBV-ACLF

**Figure 4:**
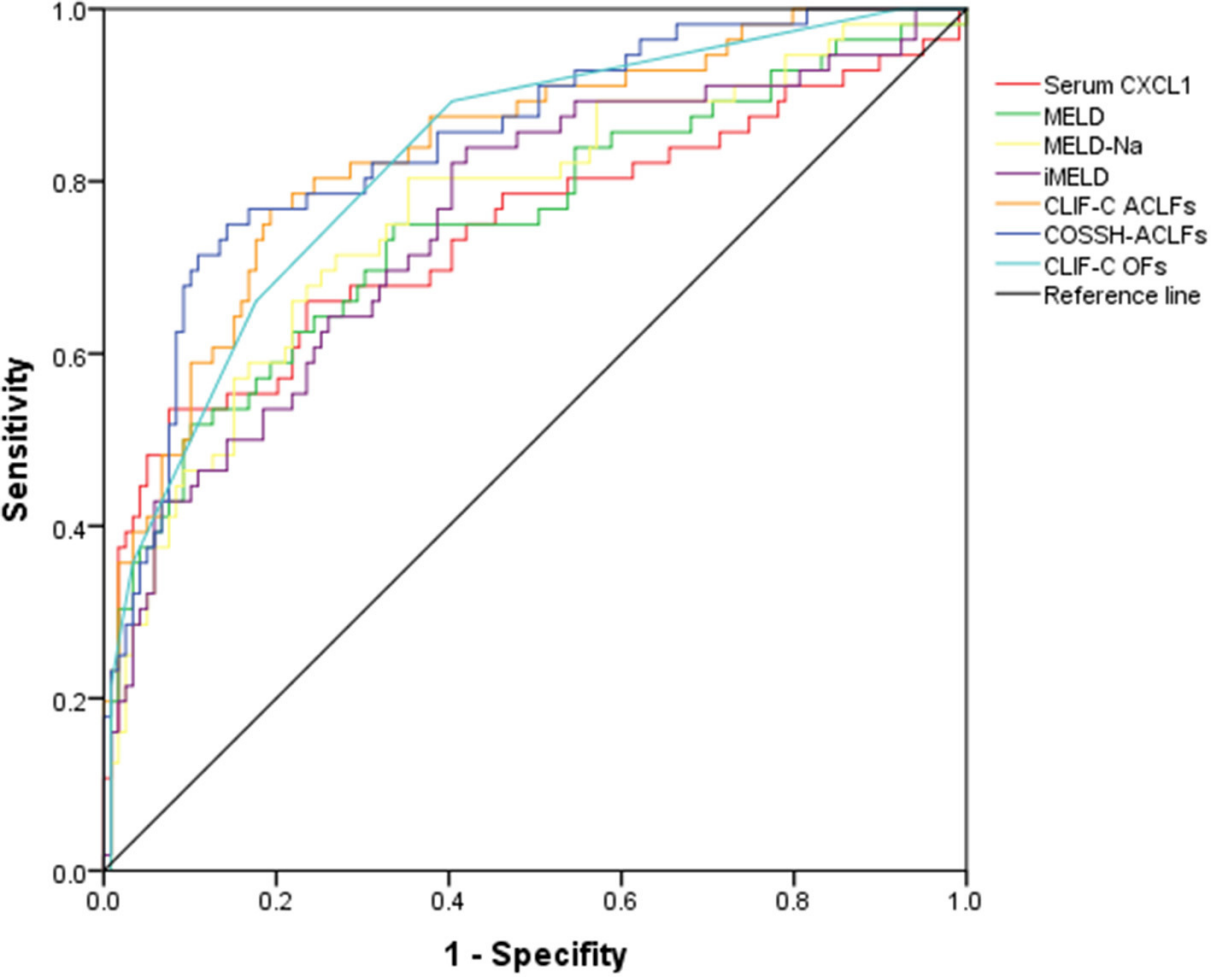
AUROC curves of prognostic models in predicting 28-day mortality in HBV-ACLF groups. AUROC, area under the receiver operating characteristic; HBV-ACLF, hepatitis B virus-related acute-on-chronic liver failure; CXCL1, C-X-C motif chemokine ligand 1; MELD, Model for End-Stage Liver Disease; MELD-Na, MELD-sodium; iMELD, integrated MELD; CLIF-C ACLFs, CLIF-Consortium-ACLF score; COSSH-ACLF score, Chinese Group on the Study of Severe Hepatitis B-ACLF; CLIF-C OFs, CLIF-Consortium Organ Failure Score.

We conducted AUROC analysis in patients with HBV-ACLF (*n* = 175) comparing serum CXCL1 with other prognostic scores. Serum CXCL1 exhibited strong prognostic ability, with a high AUROC value [0.741 (0.669–0.804)] for predicting 28-day mortality in patients with HBV-ACLF, which was comparable to the AUROC values obtained with the MELD score [0.750 (0.679–0.812)], the MELD-Na score [0.765 (0.695–0.826)], the iMELD score [0.753 (0.683–0.815)], the CLIF-C OFs [0.825 (0.760–0.878)] and the CLIF-C ACLFs [0.836 (0.773–0.888), *P* > 0.05 for all comparisons; Figure 4 and Table 4].

**Table 4:** Comparison of the predictive value of prognostic scoring systems for patients with HBV-ACLF on 28-day survival.

**Scores**	**AUROC (95%CI)**	**Z value**	***P*-value**
**Derivation (** ***n*** **=** **175)**			
Serum CXCL1	0.741 (0.669–0.804)		
MELD	0.750 (0.679–0.812)	0.148	0.8824
MELD-Na	0.765 (0.695–0.826)	0.424	0.6714
iMELD	0.753 (0.683–0.815)	0.228	0.8193
CLIF-C OFs	0.825 (0.760–0.878)	1.586	0.1127
CLIF-C ACLFs	0.836 (0.773–0.888)	1.731	0.0834
COSSH-ACLFs	0.846 (0.783–0.896)	2.035	0.0418

*The AUROCs for different models were calculated and compared using the Z test (Delong's method). HBV-ACLF, hepatitis B virus-related acute-on-chronic liver failure; auROC, area under the receiver operating curve; CXCL1, C-X-C motif chemokine ligand 1; MELD, model for end-stage liver disease; MELD-Na, MELD-sodium; iMELD, integrated MELD; CLIF-C ACLFs, CLIF-consortium-ACLF score; COSSH-ACLF score, chinese group on the study of severe hepatitis B-ACLF; CLIF-C OFs, CLIF-consortium organ failure score*.

## Discussion

In liver disease, CXCL1 has been found to have a role in alcoholic liver injury, steatohepatitis, and HCC (14–16), while previous studies have not examined the relationship between CXCL1 and ACLF. Our study found that expression of CXCL1 was dramatically increased in patients with HBV-ACLF as compared to that in healthy controls and patients with CHB and HBV-CC. Serum CXCL1 at admission was also significantly higher in nonsurvivors than in survivors with HBV-ACLF, demonstrating that CXCL1 can serve as an important indicator of short-term survival in patients with HBV-ACLF.

An excessive systemic inflammatory response seems to play a crucial role in the development of ACLF (1). Neutrophils are a hallmark of activation of the innate immune system (26), and in various experimental models activated neutrophils can induce liver injury (27, 28). As the effector cells of acute inflammation, neutrophils have long been reported to have a role in the maintenance of homeostasis and in the pathogenesis of liver injury. Neutrophil dysfunction has been reported in patients with ACLF (29). Makkar et al. suggested that neutrophil dysfunction may could predict 90-day survival in patients with ACLF (30). The CXC family of chemokines attract neutrophils and lymphocytes, which are activated by binding of these chemokines to appropriate receptors (31). As a neutrophil marker, CXCL1 binds to its receptor CXCR2, playing a crucial role in the host immune response by recruiting and activating neutrophils and inducing neutrophil migration (32). Thus, identification of novel neutrophil biomarkers that enable early and accurate detection of neutrophil dysfunction is needed for timely prognosis of patients with HBV-ACLF.

In mice with CCL4-induced liver fibrosis, up-regulated CXCL1 expression in hepatic stellate cells has been reported to promote it activation and modulate liver fibrogenesis (33). However, we found the serum CXCL1 levels in healthy controls and patients with CHB and HBV-CC were similar. Further, several lines of evidence indicate that neutrophils are major effectors of acute inflammation, can be actively recruited during acute-phase reactions, and act as a marker of disease severity and tissue inflammation (9). Our study revealed that patients with HBV-ACLF had significantly higher neutrophil count than healthy controls and patients with CHB and HBV-CC, besides the neutrophil count was correlated with HBV-ACLF severity. Consistent with the results for CXCL1, no difference in neutrophil count was found among healthy controls and patients with CHB and HBV-CC. In a parallel study, Deng et al. showed that patients with HBV-related decompensated cirrhosis had a significantly higher neutrophil-to-lymphocyte ratio than patients with CHB and HBV-CC (9). CHB is characterized by chronic inflammatory responses and, therefore, chronic, mild HBV infection may not result in a significant increase in serum CXCL1 levels.

Patients with ACLF experience highly dynamic changes over the course of hospitalization. Most patients receive a preliminary prognosis 3 to 7 days after hospital admission based on common ACLF prognostic scores, including MELD, CLIF-C OFs, and CLIF-C ACLFs, among others (1). Clinical decisions such as whether to perform liver transplantation or place a patient on an artificial liver support system are made accordingly. Currently, no study has directly analyzed the prognostic effect of CXCL1 in populations with HBV-ACLF. Our study showed that serum CXCL1 is closely related to HBV-ACLF severity. We further analyzed the prognostic ability of CXCL1 in our cohort, allowing for comparison of CXCL1 and various prognostic scores. Except for COSSH-ACLFs, CXCL1 was comparable to common ACLF prognostic scores (MELD, MELD-Na, iMELD, CLIF-C OFs, and CLIF-C ACLFs). We showed that high serum CXCL1 is a strong indicator in prognosis of patients with ACLF, and that alleviating neutrophil dysfunction in these patients may improve short-term survival.

Several studies have indicated that CXCL1 has critical roles in growth and apoptosis and may, thus, be a potential molecular target for use in HCC therapy (12). The COSSH population study indicated that INR was the most important risk factor associated with 28-day mortality in HBV-ACLF. In our study, univariate logistic regression analysis showed that without considering values of INR or other parameters in patients with HBV-ACLF, high levels of CXCL1 alone were correlated with a significantly higher risk of death at 28 days after initial hospital admission. Multivariate logistic regression analysis revealed that high CXCL1, along with older age, high INR, and serious HE, was an independent risk factor for 28-day mortality in patients with HBV-ACLF. Therefore, CXCL1 might also be a therapeutic target in HBV-ACLF management.

In the population of all patients with HBV-ACLF, high levels of CXCL1 were closely correlated with coagulation and brain failure; however, no association was found between higher CXCL1 levels and liver, kidney, respiratory system, or circulatory system failure. This may be due to inherent bias in the study population. As few patients presented with kidney, respiratory system, or circulatory system failure and most patients exhibited liver failure, it was difficult to identify a correlation between CXCL1 and organ failure in these other systems.

Our study had several limitations. First, this was a modest-sized case series of patients admitted to our hospital; collection of additional data for a larger population will help to alleviate inherent bias and further define the performance of CXCL1 as a predictive marker. Second, a cut-off value for CXCL1 is necessary to stratify patients, which can help guide clinical treatment. The cut-off for serum CXCL1 levels should be further determined in larger studies. Third, ACLF is a syndrome characterized by acute decompensation of chronic liver disease. Chronic viral hepatitis and alcohol use are the most common underlying liver diseases. While an excessive systemic inflammatory response seems to play a crucial role in the development of ACLF, we have explored the role of CXCL1 only in HBV-ACLF, and studies in ACLF of other etiology are also warranted.

## Data Availability Statement

The raw data supporting the conclusions of this article will be made available by the authors, without undue reservation.

## Ethics Statement

The studies involving human participants were reviewed and approved by the Ethics Committee of the First Affiliated Hospital, Zhejiang University School of Medicine. The patients/participants provided their written informed consent to participate in this study.

## Author Contributions

LL designed the study. LX, LZ, SM, and FZ collected the blood samples. LX and ST collected the clinical data and analyzed it and wrote first draft of the report. YZ and ZX are responsible for reviewing. All authors contributed to the article and approved the submitted version.

## Conflict of Interest

The authors declare that the research was conducted in the absence of any commercial or financial relationships that could be construed as a potential conflict of interest.

## Publisher's Note

All claims expressed in this article are solely those of the authors and do not necessarily represent those of their affiliated organizations, or those of the publisher, the editors and the reviewers. Any product that may be evaluated in this article, or claim that may be made by its manufacturer, is not guaranteed or endorsed by the publisher.

## Abbreviations

ACLF: acute-on-chronic liver failure
CXCL1: Chemokine (CXC) ligand 1
HBV-ACLF: hepatitis B virus–related ACLF
CHB: chronic hepatitis B
HBV-CC: HBV-related compensated cirrhosis
AUROC: area under the receiver operating characteristic
HBsAg: HBV surface antigen
CXCR2: CXC receptor 2
HCC: hepatocellular carcinoma
HE: hepatic encephalopathy
ALT: alanine aminotransferase
AST: aspartate aminotransferase
TBIL: total bilirubin
COSSH-ACLF: Chinese Group on the Study of Severe Hepatitis B–ACLF
CLIF-C OFs: CLIF Consortium Organ Failure Score
MELD: Model for End-Stage Liver Disease
MELD-Na: MELD-sodium
iMELD: integrated MELD
CLIF-C ACLFs: CLIF Consortium ACLF score.
