# Supplementary material for: Serum CXCL1 Is a Prognostic Factor for Patients With Hepatitis B Virus–Related Acute-On-Chronic Liver Failure
Source: Front Med (Lausanne). 2021 Jul 28;8:657076. doi: 10.3389/fmed.2021.657076 (PMC8355541; doi:10.3389/fmed.2021.657076)
Supplement: Supplementary file 1 [file Data_Sheet_1.docx]

Supplementary Material

# Supplementary Methods

## Scoring

The MELD score was calculated as MELD = 9.57 $\times$ln[creatinine (mg/dl)] + 3.78$\times$ln[TBIL (mg/dl)] + 11.2$\times$ ln(INR) + 6.4 ([1](#_ENREF_1)).

The MELD-Na score was calculated as MELD-Na = MELD + 1.59$\times$(135 – Na) ([2](#_ENREF_2)).

The iMELD score was calculated as iMELD = MELD + 0.3$\times$age (years) ­– 0.7$\times$Na (mmol/liter) + 100 ([3](#_ENREF_3)).

CLIF-C OFs was used for identification of organ failure ([4](#_ENREF_4)).

CLIF-C ACLFs was calculated based on the CLIF-OFs as follows: CLIF-C ACLFs = 10$\times$[0.33$\times$CLIF-C OFs + 0.04 $\times$age (years) + 0.63 $\times$ln(white blood cell count) ­– 2] ([4](#_ENREF_4)).

# COSSH-ACLFs was calculated as COSSH-ACLFs = 0.741 $\boldsymbol{\times}$INR + 0.523 $\boldsymbol{\times}$HBV-SOFA + 0.026$\boldsymbol{\times}$age (years) + 0.003$\boldsymbol{\times}$TBIL ([5](#_ENREF_5)).References

1. Kamath PS, Kim WR. The model for end-stage liver disease (MELD). *Hepatology*. (2007) 45:797-805. doi: 10.1002/hep.21563

2. Biggins SW, Kim WR, Terrault NA, Saab S, Balan V, Schiano T, et al. Evidence-based incorporation of serum sodium concentration into MELD. *Gastroenterology*. (2006) 130:1652-60. doi: 10.1053/j.gastro.2006.02.010

3. Luca A, Angermayr B, Bertolini G, Koenig F, Vizzini G, Ploner M, et al. An integrated MELD model including serum sodium and age improves the prediction of early mortality in patients with cirrhosis. *Liver Transpl*. (2007) 13:1174-80. doi: 10.1002/lt.21197

4. Jalan R, Saliba F, Pavesi M, Amoros A, Moreau R, Ginès P, et al. Development and validation of a prognostic score to predict mortality in patients with acute-on-chronic liver failure. *J Hepatol*. (2014) 61:1038-47. doi: 10.1016/j.jhep.2014.06.012

5. Wu T, Li J, Shao L, Xin J, Jiang L, Zhou Q, et al. Development of diagnostic criteria and a prognostic score for hepatitis B virus-related acute-on-chronic liver failure. *Gut*. (2018) 67:2181-2191. doi: 10.1136/gutjnl-2017-314641
